# Effect of Resident and Fellow Involvement on Outcomes of Sarcoma Surgery: A NSQIP Database Cross-Sectional Study

**DOI:** 10.1155/2021/2645737

**Published:** 2021-12-18

**Authors:** Eugene S. Jang, Michael G. Artin, Venkat Boddapati, Chung Min Chan, Andre R. Spiguel, C. Parker Gibbs, Mark T. Scarborough, Wakenda K. Tyler

**Affiliations:** ^1^University of Florida, Department of Orthopaedics and Rehabilitation, 3450 Hull Road, Gainesville, FL 32607, USA; ^2^Columbia University Medical Center, Department of Orthopaedic Surgery, 622 West 168th Street PH11, New York, NY 10032, USA

## Abstract

**Background:**

The complexity of sarcoma surgery often justifies surgical assistants of higher levels of academic training: senior residents, fellows, or co-surgeons. The association between the level of training of assistants and outcomes of these procedures has yet to be studied.

**Methods:**

The Current Procedural Terminology (CPT) codes comprising the “core” procedures for musculoskeletal oncology fellowships were gathered. After CPTs primarily capturing nononcologic procedures were excluded, the National Surgical Quality Improvement Program (NSQIP) database was used to find procedures with these CPTs. The severity of complications was assessed using the Severity Weighting of Postoperative Adverse Events in Orthopedic Surgery (SWORD) score. Resident/fellow presence was analyzed both as a binary variable and stratified by level of training.

**Results:**

In 159 cases meeting inclusion criteria, higher-level assistants were associated with increased rate of any complication (*p*=0.006) and greater need for transfusion (*p*=0.001) but also tended to be used in cases of longer duration (*p*=0.001) and with higher total work relative value units (wRVUs) (*p*=0.001). Multivariate analysis showed that while higher-wRVU procedures persisted as an independent predictor of increased complications (OR 1.028 per RVU unit, *p*=0.002), neither the presence nor level of training of assistants had an independent effect on complication rates. Other independent predictors of 30-day complications were treatment comorbidity (OR 3.433, *p*=0.010) and lower extremity location of the tumor (OR 4.393, *p*=0.006). Severity of complications did not differ between any of the groups on either univariate or multivariate analysis.

**Conclusions:**

Trainees of higher levels of academic training tend to be present for longer, higher-complexity musculoskeletal oncology cases, but the overall severity of complications from these do not significantly differ from lower-risk cases without trainees. Orthopedic oncologists may reassure patients that the presence of trainees and co-surgeons is not only safe but it may also help reduce the severity of complications in more complex procedures.

## 1. Introduction

Surgeons and patients may wonder alike how the presence of residents and fellows in their surgeries ultimately affects the outcomes of the procedure. Previous studies in other surgical specialties have come to various conclusions on this matter, ranging from worse outcomes when trainees are present [1–3], to no effect [4], to an overall benefit [5, 6]. Within orthopedic surgery specifically, a recent systematic review of 22 studies examining common orthopedic procedures concluded that resident involvement was associated with minor increases in morbidity following complex procedures, but no differences in overall patient mortality [7]. This review, however, did not include any procedures within the realm of orthopedic oncology.

The practice of orthopedic oncology, by nature of its multidisciplinary approach and patient factors, often takes place in academic institutions where residents and fellows are involved in patient care. Patients may have substantial perioperative risk due to the effects of chemotherapy or radiation; furthermore, the extensive nature of these procedures and the vascularity of tumors can lead to a higher risk of bleeding [8]. The complexity of the operative procedures performed by musculoskeletal oncologists may at times serve as justification for the involvement of more experienced operating room staff, surgical assistants of higher levels of postgraduate training, and other orthopedic and nonorthopedic faculty to serve as co-surgeons. The association between the presence and level of training of surgical trainees and outcomes after surgery has yet to be studied systematically in the subspecialty of musculoskeletal oncology.

The aim of this study is to elucidate the association between the presence of trainees during orthopedic oncology surgery and perioperative complications. In a subspecialty where high-risk patients and procedures are common, it becomes especially relevant to characterize how the personnel in the operating room can affect the potential risks of surgery. The complexity of these types of surgery often serves as justification for requiring assistants with higher levels of academic training, and this study aims to answer the question of whether this rationale stands to reason.

The questions to be addressed by this study are as follows:

1. For the core surgical procedures that are primarily performed by orthopedic oncologists, is there an association between the presence/level of training of trainees and the rate of complications after surgery?
2. On multivariate analysis, does the presence/level of training of trainees persist as independent predictors of the rate of complications?
3. Does the presence/level of training of trainees have an effect on the relative severity of complications?

## 2. Patients and Methods

### 2.1. Study Design and Participants

The Current Procedural Terminology (CPT) codes corresponding to the “relevant oncologic procedures” put forth for musculoskeletal oncology fellowships by the Accreditation Council for Graduate Medical Education (ACGME) Case Log Guidelines were gathered [9]. This selection of procedures comprises the list of “case log minimums” for musculoskeletal oncology fellowships and aims to cover the full spectrum of core procedures that are central to the practice of tumor surgery. The list of CPTs was narrowed to exclude those deemed likely to capture a significant proportion of nononcologic procedures (e.g., open treatment of fractures and arthroplasty), leaving the following set of CPTs: (22101, 22102, 23210, 23220, 24150, 25170, 27075, 27076, 27077, 27365, 27645, 27646, 27647). These decisions were made by two of the authors independently (EJ, VB) with any differences adjudicated by the senior author (WT) in order to minimize selection bias.

The American College of Surgeons' National Surgical Quality Improvement Program (ACS NSQIP) database, which has been used extensively for large-scale clinical research, was used to find a convenience sample comprised of procedures with these CPTs during those years in which the ACS also collected data on assistant level of training (2005–2012) [10–13].

Of the 462 records with data available on assistant level of training and excluding those records with missing baseline patient characteristics (*n* = 19), 443 patient records remained from all institutions. The International Classification of Diseases Ninth Revision (ICD-9) codes of the remaining patient records were examined for conditions unrelated to orthopedic oncology and those procedures excluded (e.g., if an endoprosthesis reconstruction was performed for trauma purposes). The ICD-9 codes included comprised both benign and malignant primary lesions of bones and connective tissue: 162.9, 170.^*∗*^, 171.^*∗*^, 189.0, 195.3, 195.5, 198.5, 203.00, 213.^*∗*^, 214.^*∗*^, 215.2, 238.^*∗*^, 239.2, 727.02, 733.22, 733.29. Decisions about inclusion were made by two of the authors independently (EJ, VB) with any differences adjudicated by the senior author (WT). After applying ICD-9 code inclusion and exclusion criteria, 159 total cases remained for the analysis.

Data on patient comorbidities, minor and major complications within 30 days, total operative time in minutes, and work relative value units (wRVUs) assigned to the procedure were extracted from the NSQIP database. Resident and fellow involvement was assessed as a binary measure (trainees present or absent) and also stratified by the level of training of assistant by postgraduate year (PGY) level, into junior resident (PGY 1–3), senior resident (PGY 4-5), and fellow/co-surgeon (PGY 6+) strata.

### 2.2. Statistical Analysis

For the unadjusted analysis, baseline and perioperative variables tracked in the ACS NSQIP database were collected and stratified by resident presence, with a 0 in the PGY variable representing “no resident/fellow” and any higher number representing “resident/fellow.” Composite measures of patient comorbidities were collected including cardiac, pulmonary, neurologic, and hematologic comorbidities, as well as preoperative chemotherapy or radiation. Outcomes were classified into “medical complications” and “surgical complications” using groupings used in previously published research on this subject [5, 14]. “Any complication” was defined as the occurrence of either a “medical complication” or a “surgical complication.” These complications, along with unplanned readmissions, unplanned reoperations, death, operative time, and total length of hospital stay, were compared between groups using the Wilcoxon rank-sum tests for continuous variables and Fisher's exact tests for categorical variables.

For multivariate regression, a multiple logistic regression was performed for the outcome of “any complication.” The following variables were identified from clinical expertise and the literature as being prognostic and/or potential confounders [15] for complications in orthopedic oncology surgery: older age, hematologic comorbidity, preoperative chemotherapy or radiation, higher American Society of Anesthesiologists (ASA) class, total work relative value units (wRVUs), [16] and lower extremity surgery (including the pelvis) [8, 17]. Penalized maximum likelihood using the Firth method was utilized in order to reduce small-sample bias and avoid problems related to data separation [18].

For severity of complications, a secondary analysis was performed to assess for difference in severity of complications based on resident presence. Complication severity was compared between groups using the Severity Weighting of Postoperative Adverse Events in Orthopedic Surgery (SWORD) score, a validated outcomes score specifically designed to assess severity of complications on a scale of 0% (no complication) to 100% (death) [19]. The score reports less severe complications represented by lower numbers (e.g., urinary tract infection at 0.23%) and more severe complications by higher numbers (e.g., coma, 15.14%). Patients are assigned a score equal to the most severe adverse event they experienced. Because the SWORD score is a proportion, fractional logistic regression was used both for univariable and adjusted multivariable analyses to assess impact of resident participation on complication severity [20].

Statistical significance was set at *p* < 0.05. All analysis was performed with Stata 16.1/SE (StataCorp LLC, College Station, TX, USA). Data are presented as mean ± standard deviation, odds ratio (OR) with 95% confidence intervals (CI), or beta coefficient with *p* values, as appropriate. The STROBE statement-checklist for cross-sectional studies was used.

### 2.3. Patient Population

A total of 159 cases met the inclusion criteria between 2006 and 2012. Baseline medical characteristics were similar amongst the groups, including age, race, cancer stage, and preexisting comorbidities (Table 1).

## 3. Results

Univariate analysis of resident/fellow presence versus the rates of 30-day surgical and medical complications was performed (Table 2). Of note, the presence of a resident or fellow was associated with a higher incidence of “any complication” (40% vs. 16.7% without, *p*=0.037), and a significantly higher rate of blood transfusion within 30 days of surgery (31.9% vs. 8.3%, *p*=0.025) when a resident or fellow was present. Presence of trainees was associated with cases of higher complexity, as evidenced by an average wRVU level of 49.53 in cases with trainees vs. 36.29 without (*p*=0.038). Procedures with a resident or fellow present were also associated with longer average case duration (208.39 minutes with trainees vs. 125.42 minutes without, *p*=0.003) as well as with a longer mean length of hospital stay (6.30 days with trainees vs. 3.41 days without, *p*=0.006). When the level of training was taken into consideration, there was a direct correlation found between higher assistant training level and increased rate of any complication (*p*=0.006) as well as increased need for transfusion (*p*=0.001). The presence of higher level was directly correlated with longer and more complex cases, with total operative time proportionally increasing with the assistant level of training (ranging from an average of 171 minutes for cases with no resident, to 209 minutes with PGY 1–3, to 249 minutes with PGY 4-5, to 346 minutes with a PGY6+ (*p*=0.001)) and similarly with total wRVUs for the procedure (36.29 with no residents/38.27 with PGY 1–3/50.24 with PGY 4-5/59.94 with PGY6+ (*p*=0.001)).

Multivariate analysis (Table 3) revealed that the rate of total complications was independently predicted by three main factors: treatment comorbidity (OR 3.433 (95% CI 1.340–8.795, *p*=0.010)), the presence of a lower extremity (including the pelvis) tumor (OR 4.393 (95% CI 1.525–12.65, *p*=0.006)), and increased wRVU associated with the procedure (OR 1.028 per RVU unit (95% CI 1.011–1.046, *p*=0.002)). The presence of residents and fellows, however, did not persist as a significant predictor of total complications (OR 2.336 (95% CI 0.636–8.586, *p*=0.201)) after adjusting for confounding factors. Patient age, hematologic comorbidities, and ASA class of the patient also did not persist as significant predictors of complications on multivariate analysis.

**Figure 1:**
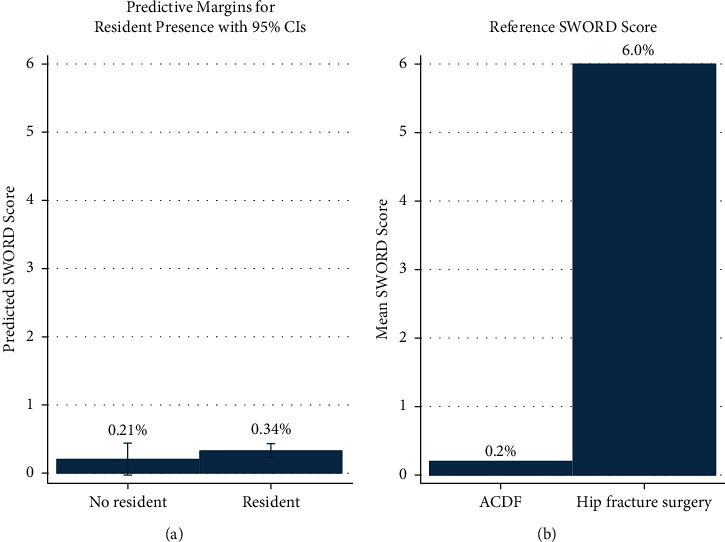
Predicted complication severity for orthopedic oncology procedures without and with resident/fellow trainees (a). As a point of comparison, the average complication severity for the procedures with the lowest and highest average SWORD score in the literature (anterior compression, decompression, and fusion (ACDF) of the spine and hip fracture surgery, respectively) (b).

When severity of complications was taken into account, no significant difference in the mean severity of complications was found between groups on either univariate or multivariate analysis. Results of univariable fractional logistic regression of the SWORD score showed no significant effect of resident presence on complication severity (*β* = 0.711, *p*=0.243) (Table 4). The mean SWORD score was 0.35% for the trainee group and 0.17% for the without trainee group. Results of multivariable fractional logistic regression likewise showed no significant effect of resident presence on complication severity (*β* = 0.490, *p*=0.415) after adjusting for the same confounding factors as the complication rate analysis. The multivariable model predicts a SWORD score of 0.34% (95% CI 0.23–0.44) with a trainee present and 0.21% (95% CI −0.02–0.44) with no trainee present (Figure 1). For reference, the procedures with the lowest (elective anterior cervical decompression and fusion) and highest (hip fracture surgery) mean SWORD scores reported in the literature are 0.2% and 6.0%, respectively.

## 4. Discussion

On univariate analysis, there was an association found between the presence of residents and fellow trainees and an increased rate of overall complications and blood transfusions within 30 days of surgery. These findings mirror the results of previous research investigating the impact of resident participation in complex surgical oncology cases, which have shown resident participation to be associated with an increased 30-day postoperative morbidity and an increased rate of hematologic complications [2, 3]. However, the surgical complexity of the procedures where residents and fellows were present was significantly higher than that without trainees and proportionally increased with increasing level of training of assistants (36.29 with no residents/38.27 with PGY 1–3/50.24 with PGY 4-5/59.94 with PGY6+, *p*=0.001). This finding suggests that the complexity of surgical cases may in fact be driving the utilization of assistants of higher levels of training.

When a multivariate analysis is performed, a clearer picture begins to arise as to the association between trainee involvement and complications. Upon accounting for confounders, resident/fellow involvement fell away as an independent predictor of complication rate. This suggests that rather than the presence of these assistants being the driving force behind higher complication rates, factors inherent to the patients and procedures are resulting in both the involvement of higher-level trainees and higher complication rates.

Interestingly, patient comorbidities and age were also variables that did not persist as significant predictors of complications on multivariate analysis, leaving tumor characteristics and the nature of treatment as the remaining possibilities for the driving factors. Indeed, preoperative chemotherapy/radiation remained as a significant predictor, as did a lower extremity location of tumor, as well as higher case complexity.

These findings together suggest that those patients who require preoperative chemotherapy/radiation—and indicated for a longer and more complex surgical interventions—are more likely to end up in an operating room where senior residents, fellows, or co-surgeons are present. As a result of their need for more complex procedures, they end up having longer surgeries with more complications, requiring more blood transfusions during and after surgery, and staying in the hospital longer. Thus, the presence of residents/fellows is not independently driving up complication rates; residents and fellows are simply being used in more complicated cases.

If residents and fellows do not significantly affect the rate of complications, the question remains as to whether their use in the more complex procedures is justified. Studies in other surgical specialties have suggested that having residents and fellows involved in procedures may reduce the relative severity of complications [5, 6]. Reassuringly, despite the higher rate of complications found in cases with assistants, there was no significant difference found in the average severity of complications. Regardless of whether resident/fellow involvement was treated as a binary variable or stratified by level of training—and whether univariate or multivariate analysis was performed—there was no impact found on severity of complications found. This is in the context of a study population consisting of very complex cases, with a mean total wRVUs per case in our sample of 47.5, compared to mean wRVUs of 21.24 for primary total hip arthroplasty and 30.27 for revision total hip arthroplasty in NSQIP [21].

This finding is in concordance with other studies of high-complexity surgical procedures. In a study of 266,411 patients who underwent high-complexity procedures (defined by high mean wRVUs, ranging from 46.6 to 52.2, in the specialties of general surgery, cardiothoracic surgery, neurosurgery, and vascular surgery), resident participation was associated with higher 30-day mortality and morbidity on unadjusted analysis. After propensity-score matching, however, there was an only a small increase in composite morbidity associated with resident presence and a significant improvement in failure-to-rescue, meaning an improved ability to avoid clinically significant deterioration such as death and disability. Outcomes were found to be improved with higher-level residents involved in the highly complex cases as well [16]. In other words, small complications may be more common in cases with resident involvement, but operative mortality and serious complications are reduced, a phenomenon which seems to be amplified in higher-complexity procedures.

### 4.1. Limitations and Future Directions

Many of the limitations of this study are inherent to other similar studies of resident and fellow involvement utilizing NSQIP [1–6]. Data in the NSQIP database may exhibit inaccuracies or selection biases, although this risk is mitigated by the use of trained reviewers, regular audits of accuracy, and multiple studies demonstrating its validity [11, 13]. Because the NSQIP database is not cancer-specific, there was no information available regarding tumor size/grade, quality of resection margins, and complications after 30 days (e.g., local recurrence and late implant failure). There is also no way to decode how much “involvement” truly takes place when a resident or fellow is recorded as having participated in a case, and the degree of involvement may differ depending on the institution, case, and personnel involved. With these studies, it is also impossible to measure the role that physician assistants (PAs) play in orthopedic oncology procedures. While the use of PAs in orthopedic oncology may be relatively rare compared to other subspecialties, there are certainly institutions in which PAs can provide an invaluable level of expertise as assistants but the NSQIP database does not have a mechanism for capturing this effect.

The association between the surgical effort required to perform a procedure and the RVUs assigned to it is certainly a controversial one; however, the measure of wRVUs has the benefit of being a predetermined and systematic measure of complexity and has been used as a proxy for surgical complexity in similar studies [16, 22]. The SWORD score is also an inherently subjective measure—as is any quantitative scoring system that ranks the relative morbidity of complications—but the SWORD score was developed systematically using a sample of orthopedic surgeons with comprehensive representation from each subspecialty.

Finally, the nature of these types of studies is to have relatively low sample sizes, due to the limited number of procedures in the database which have accurately recorded data on the level of assistants as well as the limited numbers of years during which this type of data was collected. Orthopedic oncology cases are also uncommon to begin with—comprising only ∼2% of the total caseload of orthopedic surgery residents—with substantial variability between residents and institutions [23]. This small sample size increases the challenges of statistical analysis, with the number of confounders studied via regression having to be narrowed in an effort to preserve statistical validity. Future studies comparing the magnitude of the effect of trainee presence outcomes in orthopedic oncology procedures versus those of other procedures would be helpful in assessing external validity of these methods. However, it is reassuring that the conclusions of this study are concordant with other similar studies: while the presence of residents and other trainees may potentially increase the rate of minor complications, major complication rate and severity are not significantly different.

Future studies should look to utilize institutional data sets in a multicenter collaborative study which may have more baseline variables and information about complications specific to orthopedic oncology, as well as longer follow-up and more detailed information about co-surgeons, in order to offer more detailed conclusions. While none of the other major cancer databases currently collects data about resident involvement, a multicenter retrospective study would potentially allow for greater fidelity of data. For instance, such a study may be able to better collect data about the level of involvement of assistants, better distinguish fellows versus co-surgeons, and allow for collection of more oncologic outcome data over a longer term of follow-up.

## 5. Conclusions

In this subset of procedures specific to musculoskeletal oncology, the presence of residents/fellows is not associated with an increased risk of 30-day complications when accounting for confounders. Multivariate analysis reveals that it is the increased utilization of such assistants in cases of longer duration and higher complexity and in patients with more comorbidities that appears to be driving an increased rate of complications, rather than the presence of the trainees per se. Even though residents and fellows and were disproportionally used in higher-risk cases, the overall severity of complications in these cases did not significantly differ from the lower-risk cases without trainees. These findings are concordant with other studies of high-complexity and high-risk surgeries, where having more experienced assistants seems to help mitigate the risk of higher-severity complications such as death and permanent disability. Orthopedic oncologists may reassure their patients that the presence of residents, fellows, and attending co-surgeons is not only safe and important for training, but it may also play a role in keeping the overall severity of complications lower in more complex procedures.

## Figures and Tables

**Table 1:** Baseline patient characteristics, stratified by trainee presence.

	No trainee	Trainee	*p* value
	*N* = 24	*N* = 135	
Age	56 (37–73)	52 (35–65)	0.34
Male sex	13 (54.2%)	81 (60.0%)	0.65
Race			0.55
White	18 (75.0%)	102 (75.6%)	
Black	2 (8.3%)	11 (8.1%)	
Asian	2 (8.3%)	4 (3.0%)	
Other and unreported	2 (8.3%)	18 (13.3%)	
Body mass index (BMI)	27.94 (5.07)	28.52 (6.18)	0.66
Hypertension	3 (12.5%)	52 (38.5%)	0.02^*∗*^
Diabetes	0 (0.0%)	14 (10.4%)	0.13
Smoker	5 (20.8%)	29 (21.5%)	1.00
Alcohol use	1 (4.2%)	1 (0.7%)	0.28
Cardiac comorbidity	1 (4.2%)	6 (4.4%)	1.00
Previous PCI	0 (0.0%)	3 (2.2%)	1.00
Previous cardiac surgery	1 (4.2%)	2 (1.5%)	0.39
History of angina	0 (0.0%)	1 (0.7%)	1.00
History of PVD	0 (0.0%)	1 (0.7%)	1.00
Pulmonary comorbidity	0 (0.0%)	8 (5.9%)	0.61
Dyspnea	0 (0.0%)	6 (4.4%)	0.59
History of COPD	0 (0.0%)	2 (1.5%)	1.00
Neurologic comorbidity	0 (0.0%)	9 (6.7%)	0.36
Hemiplegia	0 (0.0%)	1 (0.7%)	1.00
History of TIA	0 (0.0%)	1 (0.7%)	1.00
History of CVA with deficit	0 (0.0%)	2 (1.5%)	1.00
History of CVA without deficit	0 (0.0%)	2 (1.5%)	1.00
Tumor in CNS	0 (0.0%)	4 (3.0%)	1.00
Wound comorbidity	1 (4.2%)	18 (13.3%)	0.31
Preoperative wound infection	0 (0.0%)	7 (5.2%)	0.60
Wound classification other than “Clean”	1 (4.2%)	13 (9.6%)	0.70
Hematologic comorbidity	3 (12.5%)	13 (9.6%)	0.71
Bleeding disposition	2 (8.3%)	8 (5.9%)	0.65
Transfusion of ≥1 U of whole/packed RBCs in 72 hr prior to surgery	1 (4.2%)	5 (3.7%)	1.00
ASA classification	1.38 (0.58)	1.51 (0.71)	0.38
Functional status other than “Independent”	0 (0.0%)	5 (3.7%)	1.00
>10% loss of body weight in last 6 months	0 (0.0%)	8 (5.9%)	0.61
Disseminated cancer	5 (20.8%)	34 (25.2%)	0.80
Preoperative chemotherapy or radiation	5 (20.8%)	34 (25.2%)	0.80
Chemotherapy for malignancy in <=30 days pre-op	2 (8.3%)	28 (20.7%)	0.25
Radiotherapy for malignancy in <=90 days pre-op	3 (12.5%)	10 (7.4%)	0.42
Steroid use for chronic condition	0 (0.0%)	5 (3.7%)	1.00
Prior operation in <=30 days pre-op	0 (0.0%)	3 (2.2%)	1.00
Days from hospital admission to operation	1.74 (±2.47)	2.77 (±2.68)	0.35
Emergency surgery	1 (4.2%)	1 (0.7%)	0.28
Malignancy	16 (66.7%)	113 (83.7%)	0.084
Lower extremity surgery	13 (54.2%)	102 (75.6%)	0.046^*∗*^
General anesthesia	23 (95.8%)	127 (94.1%)	1.00
Total wRVUs	28.36 (±2.04)	42.51 (±1.76)	0.002^*∗*^

Data are presented as mean (SD) for “Body Mass Index,” or geometric mean (±geometric SD) for “Days from hospital admission to operation” and “Total wRVUs,” or median (IQR) for “Age,” and *n* (%) for categorical measures. Student's *t*-test was used for “Body Mass Index” and “ASA class,” the Wilcoxon rank-sum test was used for other continuous measures, and Fisher's exact test was used for categorical measures. The following baseline variables tracked by the ACS NSQIP database had no occurrences in this cohort and are excluded from the table: history of congestive heart failure, history of myocardial infarction, peripheral vascular disease with rest pain, ventilator dependence, preoperative pneumonia, preoperative coma, impaired sensorium, paraplegia, quadriplegia, and preoperative sepsis. CVA = cerebrovascular accident, TIA = transient ischemic attack, PCI = percutaneous cardiac intervention, PVD = peripheral vascular disease, COPD = chronic obstructive pulmonary disease, CNS = central nervous system, and wRVUs = work relative value units. ^*∗*^*p* < 0.05.

**Table 2:** Univariable surgical outcomes stratified by trainee presence.

	No trainee	Trainee	*p* value
	*N* = 24	*N* = 135	
Any complication	4 (16.7%)	54 (40.0%)	0.037^*∗*^
Medical complication	4 (16.7%)	49 (36.3%)	0.065
Pneumonia	0 (0.0%)	1 (0.7%)	1.00
Unplanned reintubation	0 (0.0%)	0 (0.0%)	
Pulmonary embolism	0 (0.0%)	4 (3.0%)	1.00
Acute renal failure	0 (0.0%)	0 (0.0%)	
Urinary tract infection	0 (0.0%)	1 (0.7%)	1.00
Stroke/CVA	0 (0.0%)	0 (0.0%)	
Coma	0 (0.0%)	0 (0.0%)	
Peripheral nerve injury	0 (0.0%)	1 (0.7%)	1.00
Cardiac arrest	0 (0.0%)	0 (0.0%)	
Myocardial infarction	0 (0.0%)	0 (0.0%)	
Blood transfusion	2 (8.3%)	43 (31.9%)	0.025^*∗*^
Deep vein thrombosis	0 (0.0%)	3 (2.2%)	1.00
Sepsis	2 (8.3%)	3 (2.2%)	0.16
Septic shock	0 (0.0%)	0 (0.0%)	
Surgical complication	2 (8.3%)	9 (6.7%)	0.67
Superficial incisional surgical site infection	0 (0.0%)	1 (0.7%)	1.00
Deep incisional surgical site infection	2 (8.3%)	3 (2.2%)	0.16
Organ/space surgical site infection	0 (0.0%)	2 (1.5%)	1.00
Wound dehiscence	0 (0.0%)	3 (2.2%)	1.00
Unplanned reoperation	0 (0.0%)	7 (5.2%)	0.60
Unplanned readmission	0 (0.0%)	9 (6.7%)	0.36
Death	0 (0.0%)	2 (1.5%)	1.00
Total operation time in minutes	125.42 (±2.34)	208.39 (±2.09)	0.003^*∗*^
Length of total hospital stay in days	3.41 (±2.79)	6.30 (±2.50)	0.006^*∗*^

Data are presented as geometric mean (±geometric SD) for continuous measures and *n* (%) for categorical measures. The Wilcoxon rank-sum test was used for continuous measures, and Fisher's exact test was used for categorical measures. CVA = cerebrovascular accident. ^*∗*^*p* < 0.05.

**Table 3:** Multivariable logistic regression analysis showing odds of any complication with trainee presence.

	Adjusted OR	95% CI	*p* value
Resident presence	2.336	0.636–8.586	0.201
Age	0.998	0.973–1.024	0.868
Hematologic comorbidity	2.812	0.823–9.612	0.099
Preoperative chemotherapy or radiation	3.433	1.340–8.795	0.010^*∗*^
ASA class	0.916	0.456–1.837	0.804
Total wRVUs	1.028	1.011–1.046	0.002^*∗*^
Lower extremity surgery	4.393	1.525–12.656	0.006^*∗*^

CI = confidence interval, ASA = American Society of Anesthesiologists, and wRVUs = work relative value units. ^*∗*^*p* < 0.05.

**Table 4:** Univariable and multivariable fractional logistic regression analyses of trainee participation and complication severity.

	Univariable beta (95% CI)	*p* value	Multivariable beta (95% CI)	*p* value
Trainee presence	0.711 (−0.481–1.902)	0.243	0.488 (−0.685–1.661)	0.415
Age	—	—	0.002 (−0.015–0.020)	0.791
Hematologic comorbidity	—	—	−0.141 (−1.024–0.741)	0.754
Pre-op chemotherapy/radiation	—	—	0.210 (−0.448–0.868)	0.532
ASA class	—	—	−0.237 (−0.640–0.165)	0.248
Total wRVUs	—	—	0.011 (0.006–0.017)	<0.001^*∗*^
Lower extremity surgery	—	—	0.492 (−0.571–1.555)	0.365

There was no significant association between of resident presence and severity of complications found, as measured by the SWORD score (the severity weighting of postoperative adverse events in orthopedic surgery). CI = confidence interval, ASA = American Society of Anesthesiologists, and wRVUs = work relative value units. ^*∗*^*p* < 0.05.

## Data Availability

The clinical database-derived data used to support the findings of this study are included in the article.
